# Audiovisual angle and voice incongruence do not affect audiovisual verbal short-term memory in virtual reality

**DOI:** 10.1371/journal.pone.0330693

**Published:** 2025-08-22

**Authors:** Cosima A. Ermert, Manuj Yadav, Jonathan Ehret, Chinthusa Mohanathasan, Andrea Bönsch, Torsten W. Kuhlen, Sabine J. Schlittmeier, Janina Fels

**Affiliations:** 1 Institute for Hearing Technology and Acoustics, RWTH Aachen University, Aachen, Germany; 2 Visual Computing Institute, RWTH Aachen University, Aachen, Germany; 3 Work and Engineering Psychology, RWTH Aachen University, Aachen, Germany; University of Hamburg, GERMANY

## Abstract

Virtual reality (VR) environments are frequently used in auditory and cognitive research to imitate real-life scenarios. The visual component in VR has the potential to affect how auditory information is processed, especially if incongruences between the visual and auditory information occur. This study investigated how audiovisual incongruence in VR implemented with a head-mounted display (HMD) affects verbal short-term memory compared to presentation of the same material over traditional computer monitors. Two experiments were conducted with both these display devices and two types of audiovisual incongruences: angle (Exp 1) and voice (Exp 2) incongruence. To quantify short-term memory, an audiovisual verbal serial recall (avVSR) task was developed where an embodied conversational agent (ECA) was animated to speak a digit sequence, which participants had to remember. The results showed no effect of the display devices on the proportion of correctly recalled digits overall, although subjective evaluations showed a higher sense of presence in the HMD condition. For the extreme conditions of angle incongruence in the computer monitor presentation, the proportion of correctly recalled digits increased marginally, presumably due to raised attention, but the effect size was negligible. Response times were not affected by incongruences in either display device across both experiments. These findings suggest that at least for the conditions studied here, the avVSR task is robust against angle and voice audiovisual incongruences in both HMD and computer monitor displays.

## Introduction

Experiments in auditory and cognitive sciences research are increasingly conducted in virtual reality (VR) environments [1,2]. VR can evoke a higher feeling of presence, the feeling of “being in a scene” [3], compared to computer monitor presentations [4]. Thus, VR is widely employed to create immersive environments that approximate real-life scenarios. Compared to traditional laboratory setups with computer monitors, in most cases, experiments in VR differ in terms of the visual information presented. This offers new possibilities for designing experimental environments and tasks, but also raises the question of how this added visual information affects cognitive performance. Importantly, the effect of VR on participants is not yet fully understood and appears to be highly dependent on the experimental design. Several studies have reported positive effects of VR, such as increased attention [5,6] and enhanced motivation and engagement [7,8]. However, studies have also reported disadvantages arising from using VR, since it can cause cybersickness [9] and may evoke a higher mental load [10,11].

When implementing audiovisual tasks in VR, perceived incongruences between the auditory and visual modalities can arise. This is especially relevant when the visual VR environment is not carefully crafted and aligned with the acoustic VR environment. Such an interplay of auditory and visual information has been a key aspect of cognitive research for multiple years, albeit without VR, and being based instead on computer monitor presentations. These studies have shown that coherent auditory and visual information can be integrated into a single multimodal percept [12,13 for a review], Coherence in general is a multidimensional construct, including but not limited to spatial, temporal, and semantic alignment of the auditory and visual stimuli [12]. Spatial audiovisual integration is commonly investigated in terms of the *ventriloquism effect* [14,15], which refers to the bias in auditory spatial perception toward a synchronous but spatially discrepant visual stimulus. This effect is typically investigated using brief sounds (e.g., clicks) and visual flashes presented with small spatial separations [16]. Further, audiovisual stimuli are perceived as temporally incoherent if their stimulus onset asynchrony, i.e., the temporal offset between the onset of the auditory and visual stimuli, is large enough [13]. If auditory and visual stimuli are aligned in meaning, semantic congruence is elicited [12]. A well-known example of studying semantic incongruence is the *Stroop task* [17], where participants see color words (e.g., “red”) printed in a different color (e.g., green), and are asked to name the color of the ink. Another established audiovisual incongruence effect is the *McGurk effect* [18], which is typically studied using lip movements that are presented together with congruent or incongruent syllables (e.g., “ba”, “ga”). These studies show that lip movement can alter the auditory perceived syllable, if they share the same features (e.g., “pa” and “ba”, but not “pa” and “ta”) [18].

While the incongruences listed above have mostly been studied with computer monitor presentations, there is some research investigating audiovisual congruence in VR. For example, the ventriloquism effect has been replicated in VR by Huisman *et al*. [19]. A large angular audiovisual separation has been shown to impede user experience in terms of presence, immersion, and cybersickness, while small separation angles are typically well-tolerated by participants in VR [20]. Incongruence in lip movement in terms of the McGurk effect is detected more easily in VR than in computer monitor settings [21]. Providing coherent user interaction in VR, e.g., by synchronized audiovisual virtual hand illusions [22] or sound feedback according to the user’s movements, e.g., walking [23], has been shown to increase immersion.

Notably, these studies on audiovisual incongruences in VR have primarily focused on perception-based effects, such as detectability, or subjectively perceived presence and immersion. As VR is increasingly being employed in cognitive research, it is crucial to understand whether incongruences impact cognitive functions that rely on higher-level processing of information. While it is logical to hypothesize that audiovisual incongruences beyond a certain range could impede cognitive task performance in VR, to the best of our knowledge, this has not been demonstrated yet in experimental settings.

Moreover, within the context of real-world interaction, a particularly relevant cognitive function where auditory and visual information is integrated includes listening to a talker and trying to remember what has been said. In contrast to controlled laboratory settings, everyday face-to-face interactions typically exhibit high audiovisual congruence: when a person speaks, their voice is accompanied by matching visual cues such as spatial location, lip movement, gaze direction, vocal pitch (e.g., gender), and gestures [24] - all temporally synchronized and spatially aligned. However, in VR, any of these congruences may be compromised, for instance, when the perceived voice does not match the position or gender appearance of the virtual human, the embodied conversational agent (ECA).

Hence, this study aims to investigate the extent to which certain audiovisual incongruences that are relevant to everyday interaction between two interlocutors affect cognitive task performance and subjective assessments across head-mounted display (HMD) and traditional computer monitor presentations. To examine whether audiovisual incongruences impair memory for spoken content, we implemented an audiovisual version of the verbal serial recall task [25]. This implementation includes measuring verbal short-term memory under systematically controlled audio-visual incongruencies: in the first experiment, the effect of audiovisual angle incongruences was studied, whereas the second experiment studied the effect of voice incongruence. In both studies, an HMD and a computer monitor were used as display devices to enable a direct comparison.

## Experiment 1: Audiovisual spatial incongruence

As mentioned previously, the starting point for this study is a situation common to everyday life: listening to and remembering spoken information by an interlocutor. Traditionally, the ability to rememorize and remember verbal information has been measured using the verbal serial recall task [25]. In this task, participants have to remember and recall a sequence of verbal items, e.g., a sequence of digits or words, for a short period of time. These items are typically presented unimodally, i.e., visually only (as images [26], written [27,28], or as lip-movements [29]), or auditorily only (as spoken words [27,30], like in a listening situation). For a discussion of the differences between visual and auditory digit presentations, please refer to Yadav *et al*. [31].

This task can be extended to the aforementioned scenario of listening to an interlocutor in laboratory settings where a visual representation of the interlocutor - an ECA - speaks the digit sequences in the serial recall task. This involves the digit sequences being presented audiovisually, i.e., as simultaneous lip movements and auditory items. This variation of the classic verbal serial recall is referred to as audiovisual verbal serial recall (avVSR) in the following.

**Fig 1:**
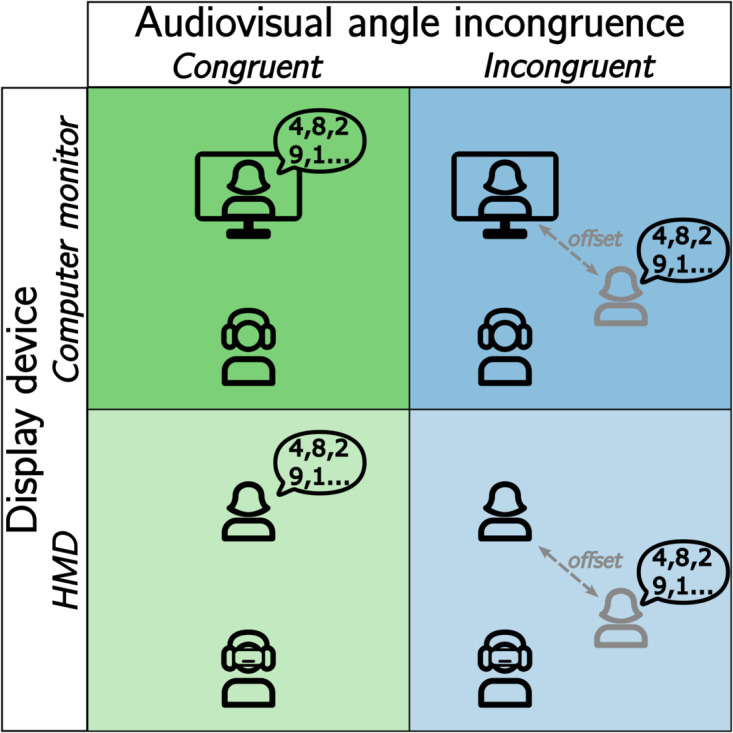
Factors in Experiment 1. The between-subject experimental design of the avVSR task with three factors: display device (computer monitor vs. HMD), the audiovisual angle incongruence (congruent vs. incongruent), and the serial position (1 - 8) of the target digit sequence. In the congruent case, the ECA and the virtual sound source are co-located; in the incongruent case, there is an angular offset between the ECA and the virtual sound source.

In selecting the audiovisual incongruence for Experiment 1, our objective was to manipulate co-verbal information conveyed by the ECA, so that the participants would naturally focus on it, while simultaneously ensuring that their ability to successfully complete the task was not directly impeded. Thus, we decided to introduce a spatial incongruence in the audiovisual stimulus presentation. More specifically, an angular offset was introduced between the visual position of the ECA and the direction from which the digit sequences were spoken to create a discrepancy between the auditory and visual scenes. The influence of this *audiovisual angle incongruence* on avVSR performance was evaluated for both HMD and computer monitor presentations as depicted in Fig 1.

### Method

#### Participants.

A total of 25 adults were recruited for the experiment via mailing lists and posters in the authors’ institutes between 27th of April and 13th of May 2022. Participants were required to be native German speakers (as the entire experiment was conducted in German), have normal hearing, and (corrected-to-)normal vision. Normal hearing below 25dB HL [32] between 250Hz and 14kHz was tested with an AURITEC Ear 3.0 audiometer and Sennheiser HDA300 headphones using a pulsed pure-tone ascending audiometry. Two participants failed the audiometry and had to be excluded. *N* = 23 participants (16 male, 6 female, 1 non-binary, aged between 19 and 36 years, *M* = 25.74, *SD* = 4.03) passed the audiometry. Normal or corrected-to-normal vision was validated with a Snellen chart up to (20/30) [33]. All participants gave written informed consent before the experiment began. Participants received a 10€ gift voucher for a bookstore. The experiment procedure was pre-approved by the Ethics Committee at the Medical Faculty of RWTH Aachen University (EK396-19). The study was conducted in accordance to the rules of conduct stated in the Declaration of Helsinki.

#### Cognitive task.

**Fig 2:**
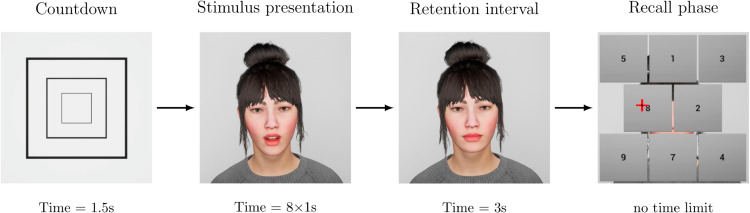
One trial of the avVSR task. Graphical depiction of the trial phases *Countdown*, *Stimulus presentation*, *Retention interval*, and *Recall phase* over time. In the *Recall phase*, the cursor is visible as a red cross.

Each experimental trial of the avVSR task was structured as follows (see Fig 2): the trial started with a visual *countdown* consisting of three rectangles decreasing in size at a rate of one rectangle per 500ms, rendered on a plane which covered the ECA entirely. Afterwards, in the *stimulus presentation phase*, participants saw the ECA speak a sequence of eight digits at a rate of one digit per second (digit word duration: 600ms, interstimulus interval: 400ms). In this phase, the ECA spoke the digits by moving its lips in accordance with the sound. The digit sequences were created from digits between one and nine in such a way that no digit was repeated and no more than two consecutive steps, up or down, occurred (e.g., “1-2-3-4” or “9-8-7-6” would not be allowed). After the stimulus presentation, participants had to wait for a *retention interval* of three seconds before they were asked to reproduce the order of the digits by clicking on the corresponding fields in a matrix, which was rendered as blocks in front of the ECA (see Fig 2). During this *recall phase*, the ECA was partially visible and animated with an idle movement. Only the eight digits present in the sequence were displayed in the matrix. The order of the digits within the matrix was changed randomly for each trial to avoid the use of visuo-spatial recall techniques [34]. Once participants clicked on a digit, it disappeared. Corrections were not possible. The dependent variables were the proportion of correctly recalled digits and the response times.

#### Apparatus and materials.

**Fig 3:**
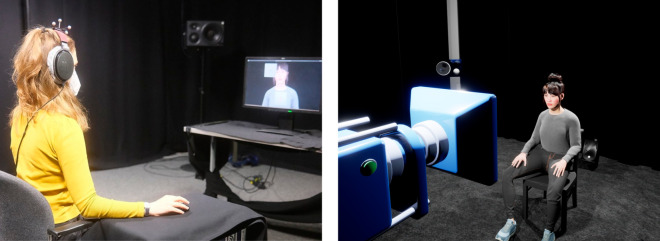
Listening experiment setup. The experiment was done with both (left) computer monitor and (right) HMD presentation. *Left*: computer monitor presentation in which a participant is seen wearing headphones with Optitrack tracking markers. *Right*: representation of the VR scene created in Unreal Engine. When wearing the HMD, the point of view of the blue camera represents the participants’ point of view.

The avVSR task was configured for both HMD and computer monitor presentations and implemented in Unreal Engine (Epic Games, v4.27) using the following plugins (see section “Supporting Information”): the StudyFramework plugin [35] (v4.26), which handles the data logging and experiment procedure, the RWTH VR Toolkit plugin [36] (v4.27) for basic VR interaction, and the Character plugin (v4.27) for ECA animation. In the HMD condition, the scene was presented using an HTC Vive Pro Eye (dual AMOLED screens with 3.5″ diagonal display, resolution 1440×1600 pixels per eye) and one HTC Vive controller. In the computer monitor condition, a Fujitsu B24T-7 (24″ diagonal display, resolution 1920 × 1080 pixels) monitor and a wireless computer mouse (Cherry MW2310) were used. To ensure consistency between the presentation modalities, the actual room in which the experiment took place (VR laboratory [37]) was replicated in VR so that the participants were virtually “in the same room” regardless of the display device used (see Fig 3).

For the visual stimulus, a female ECA (see Figs 2 and 3) was created with MetaHuman Creator (Epic Games, v0.5.2). Using Oculus Lip Sync (Meta, v20), lip animation was generated from the audio files such that the ECA moved its lips according to the digit sequence heard. In the HMD condition, the ECA sat on a virtual chair at a distance of d=2.5m from the participant, which is a comfortable inter-person distance to an unknown person [38,39]. In the computer monitor condition, only the upper part of the body was visible on the computer monitor. This image section and the distance of the computer monitor from the participants were chosen so that the size and position of the ECA were consistent between the two display devices. Participants sat on a chair, which was adjusted so that the participants (measured from the top of the head) and the ECA were approximately at the same height. The two main visual differences between display devices were that in the HMD condition the participants could not see themselves and the ECA’s lower body was not visible in the computer monitor condition.

For the acoustic stimulus, German digit words (600 ms in duration) were taken from the database by Oberem and Fels [40] (voice *female b*). The original publication did not include a recording of the digit five, but was made available by the authors. No loudspeakers were used to avoid visual fixation to possible source positions. Instead, virtual sound sources for a binaural headphone-based reproduction were auralized with Virtual Acoustics [41] (v2020a) in a reflection-free (anechoic) environment using dynamic scene rendering. Communication between Unreal Engine and Virtual Acoustics was established via the Virtual Acoustics Plugin (v4.26) (see section “Supporting Information”). This was achieved by tracking the head movements of the participants and virtually shifting the sound source position accordingly. Head-tracking was done in two different ways depending on the display device: for the HMD, the built-in tracking system was used. This was not possible in the computer monitor presentation. Instead, an OptiTrack system with Motive (OptiTrack, v2.1.1) tracked head movements. To provide plausible localization cues, the head-related transfer function (HRTF) of the Institute for Hearing Technology and Acoustics (IHTA) artificial head [42] was employed. As shown by Oberem *et al*. [43], this generic HRTF provides sufficiently accurate localization in dynamic scenes for most listeners.

The auditory stimuli were played back using Sennheiser HD650 open-back headphones with a Behringer ADA8200 sound card for both display devices. All auditory stimuli were calibrated to 60dB(A) as the power sum of both ears using the IHTA artificial head [42]. Headphone equalization was performed individually for each participant following Masiero and Fels [44].

#### Audiovisual angle incongruence.

**Fig 4:**
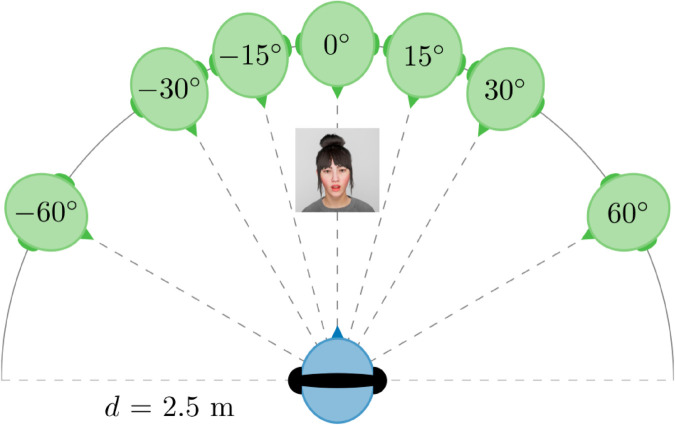
Audiovisual angle incongruence in Experiment 1. The female ECA is displayed at an azimuth angle of $0^{\circ }$ on the horizontal plane at a distance of d=2.5m from the listener (blue). The possible virtual sound source positions (green) are at the azimuth angles $0^{\circ },\pm 15^{\circ },\pm 30^{\circ },$ and $\pm 60^{\circ }$ at the distance *d*.

An angle incongruence was introduced to the audiovisual stimulus (audio: spoken digit words, visual: lip movement of ECA) by implementing an angular offset between the auditory source position and the location of the ECA. The position of the ECA as the visual source was kept constant at an azimuth angle of $\varphi =0^{\circ }$, straight ahead of the participant (see Fig 4). The idea behind the choice of the angular offsets was to include angles small enough to allow the audiovisual stimulus to be perceived as a unit and angles large enough so that an audiovisual angle incongruence is expected to be detectable. Since conversational partners are approximately at the same height in seated settings, we limited our selection to angles on the horizontal plane. Studies on the ventriloquism effect report various threshold angles up to which spatially separated visual and auditory stimuli are integrated. It is suggested that angular offset between auditory and visual source positions should not exceed $15^{\circ }$ for audiovisual integration to occur [14,45], but integration effects up to $30^{\circ }$ have been shown on the horizontal plane [46]. In their study on audiovisual congruence in VR, Kim and Lee [20] found that participants generally tolerate angular offsets of up to $30^{\circ }$ on the horizontal plane and detect angle offsets of $15^{\circ }$ only with a probability of 18.8%. It should be noted that these angular limits are usually determined with simplified audiovisual stimuli such as noise bursts and flashing lights. Thresholds for realistic sounds, such as speech, have hardly been investigated [19].

Since headphone reproduction with a generic HRTF is used here, a localization offset compared to a headphone reproduction with an individual HRTF would be expected. However, Oberem *et al*. [43] showed that this localization offset is non-significant on the horizontal plane with dynamic reproduction with head movements for the given generic HRTF with speech stimuli.

Based on the above, four angular offsets were chosen for the audiovisual angle incongruence. As a baseline condition, $\Delta \varphi =0^{\circ }$ was included, where the position of the auditory target and visual target are aligned. Furthermore, $\Delta \varphi =\pm 15^{\circ }$, $\pm 30^{\circ }$, and $\pm 60^{\circ }$ were included with decreasing probability of integration expected for increasing angular differences.

#### Questionnaires.

To link the behavioral data from the avVSR in the two display devices with subjective impressions and to quantify presence, two distinct questionnaires were used: one questionnaire that had to be completed for each display device and a second general questionnaire. Both questionnaires were implemented using SoSciSurvey in German [47].

In the first questionnaire, the perceived presence during the HMD presentation compared to the computer monitor presentation was evaluated with a German translation [48] of the Slater, Usoh, and Steed presence questionnaire (SUS) [49] on a 7-point Likert scale with the corresponding anchors. Additionally, participants were asked about the appearance of the ECA (following Ehret at al. [50]): ( **Q_Nat**: *How natural did the interlocutor seem to you?*, **Q_Spe**: *How natural did the way the interlocutor spoke seem to you?*, **Q_Sen**: *To which degree did the interlocutor appear to be a sentient being?*). Furthermore, the modality participants focused on ( **Q_Vis**: *How much did you focus visually on the interlocutor?*) and the controller handling ( **Q_Con**: *How intuitive was the handling of the controller?*) were examined.

At the end of the experiment, participants completed a second questionnaire. This questionnaire focused on the task itself ( **Q_Dif**: *How difficult was the task for you?*) and on the audiovisual incongruence ( **Q_Inc**: *Did you notice any audiovisual incongruence?* [Asked on a yes/no basis], **Q_Imp**: *If you noticed the audiovisual incongruence, did it impact your performance?*, **Q_Dom**: *If you noticed the audiovisual incongruence, did you shift your attention towards one domain (audio, visual) as a result?*). All these ratings, except for **Q_Inc**, were made on a 7-point Likert scale between 1 (minimum, e.g., “Not at all”) and 7 (maximum, e.g., “Strongly”). A comment field was provided for additional remarks. All questions and their German translation with corresponding anchors can be found in S4 File.

#### Procedure.

The experiment was conducted in individual sessions at the IHTA, RWTH Aachen University. The audiometric and visual screening was performed in a sound isolated hearing booth [37]. After passing the screening, the main experiment started in the VR laboratory [37]. The display device was varied across participants in two blocks, meaning that participants had to complete all trials on one display device before moving to the next one. The starting display device was counterbalanced between participants. At the beginning of the experiment, written instructions explaining the task were given. The participants were instructed to attend to the digit sequences and remember them, without being informed as to whether they were to prioritize the auditory or visual information. Participants were further asked not to vocalize the digits or use their fingers as an aid. The instructions were followed by a training block consisting of eight trials, two for each of the four audiovisual offsets angles ($|\Delta \varphi |=0^{\circ },15^{\circ },30^{\circ },60^{\circ }$) in counterbalanced order. The training was followed by twelve trials for each of the four angles, yielding a total of 12 × 4 = 48 trials per display device in the main experiment, divided into three blocks of 16 trials each. Each offset angle was presented twice in a block, once from the left and once from the right side; the offset angle of $|\Delta \varphi |=0^{\circ }$ was presented twice from the front. The order of angles was counterbalanced across participants to avoid order effects. Between blocks, participants could take a break of no fixed length. After each display device block, participants filled out the first questionnaire. Participants completed the second questionnaire at the end of the experiment. The reason for presenting the second questionnaire at the end rather than between the display device blocks was to avoid participants’ performance being affected by inadvertently disclosing the research questions of the listening experiment. The entire experiment, including screening and questionnaires, took approximately 90 minutes.

#### Statistical analysis.

All statistical analyses were conducted in the software R (version 4.2.2). The R package *tidyverse* [51] was used for data processing and plots. For the avVSR task, there were two dependent variables (i.e., fixed effects): the *Accuracy* and the average response times *RT_mean*. *RT_mean* is defined as the average time between two button clicks in the recall phase or, for the first digit, the time between the start of the recall phase and the first button click. *Accuracy* is the proportion of digits recalled at the correct serial position. The independent variables included the serial position, the angle (see Fig 4), and the display device. Separate Bayesian generalized mixed-effects models were created for each dependent variable using the R package *brms* (version 2.18) [52]. In each model, the repeated-measures experimental design was incorporated as independently varying intercepts (i.e., random effects) for the participants. Since *Accuracy* includes proportion data with many values close or equal to 1, a zero-one-inflated beta distribution with a logit link function was used as the family for the models with this dependent variable. A gamma distribution with a log link function was used as the family for *RT_mean* as the dependent variable since such distributions are commonly used for time data.

The modeling process for the avVSR task included starting with an intercept-only baseline model, followed by including an independent variable or an interaction between the independent variables in subsequent models. The prior distributions per independent variable and their interactions were sampled 16000 times, using four independent chains of 5000 samples each and discarding the first 1000 warm-up samples. To avoid dependencies per chain, No-U-Turn Sampling was employed. Weakly-informative prior distributions (in the logit or log scale, depending on the dependent variable) were used, which for the fixed effects included normal distributions each with a mean (*M*) of 0 and a standard deviation (*SD*) of 2. The latter is relatively large given the scales, and hence conservative. For the random effects, heavily tailed Student-t distributions with *M* = 0 and *SD* = 2 each were used. The performance across models was compared using the leave-one-out cross-validation criteria, based on the difference in values of the expected log point wise predictive density and the standard error of the expected log point wise predictive density of the models [53]. Posterior predictive checks were performed for all the models.

Pairwise comparisons across independent variable levels were conducted using the R package *emmeans* [54]. These comparisons are summarized using the median and 95% Credible Intervals (CIs) calculated using the highest density interval of the Posterior Probability Distribution (PPD). The Bayesian 95% CI provides the interval within which 95% of the PPD lies. This interval considers the probability range or the uncertainty/belief regarding the parameter value (e.g., mean), contingent on both the present sample and the prior information. This presents a more direct statement regarding the parameter than the 95% confidence interval used in frequentist statistics. The latter generally means that upon repeated random sampling and calculating the 95% confidence interval per sample, the true parameter value would be contained within approximately 95% of those intervals. This so-called long-run probability takes all values being equally likely a priori and parameter values are then contingent upon the present sample.

To determine whether an effect (comparison across independent variable levels) exists, we consider the probability of direction (PD) of the PPD, which provides the probability of an effect going in either the positive or negative direction. PD further provides a link to frequentist statistics in that the two-sided *p*-values of .05 and .01 approximately correspond to PD of 97.5% and 99.5%, respectively. PD, however, is not an index of significance. In order to consider whether an effect is meaningful/significant, we use the proportion of the 95% CI inside the region of practical equivalence (ROPE). The latter is the range signifying an effect of negligible magnitude and is conceptually similar to the null hypothesis in frequentist statistics. The range for the ROPE was specified as $\pm 0.1\times {SD}_{IV}$ [55]. PD and ROPE were calculated using the R package *bayestestR* [56].

For each of the questionnaire items, separate Bayesian mixed-effects models were created with the display device as the only fixed effect and independently varying intercepts (random effects) for the participants. For all questions except for Q_Inc, the ratings (the dependent variable) were modelled using the cumulative family with a probit link, since the ratings were on an ordinal scale. For Q_Inc, the rating was modelled using the Bernoulli family with a logit link, since the ratings were binary (yes/no). A weakly informative prior with a mean of 0 and a standard deviation of 5 was used for the dependent variables. Other modeling criteria were the same as used for the avVSR data. The differences in ratings between the display devices are summarized using the standard deviation and 95% CI (calculated using the highest density interval of the PPD) for all questions except Q_Inc, for which the change in log-odds of the response and the associated 95% CI is used.

### Results

#### Audiovisual verbal serial recall.

The full result data is given in S1 File, plots of the data can be found in S3 File. Based on the leave-one-out criteria with *Accuracy* as the dependent variable, the baseline (intercept-only) model was the worst model, while the performance across models with the various independent variables and their interactions were not substantially different. Hence, the model with all the independent variables and an interaction term between the audiovisual offset angle and display device was selected as the final model since it encompasses the experimental design most comprehensively. The interaction term with serial position was not considered useful for the research intend and hence not included. The pairwise comparison for the final model is presented in Table 1. The median differences across the comparisons were generally quite low with PD less than 97.5% except for the difference between the $0^{\circ }$ and $60^{\circ }$ angles with the computer monitor display device. The latter indicates that there is a higher *Accuracy* in the $60^{\circ }$ compared to the $0^{\circ }$ condition, suggesting an effect. However, the high percentage of the PPD within the ROPE suggests that this effect is likely of negligible practical significance (i.e., negligible effect size), as it falls within the range which is generally considered too small to be meaningful.

**Table 1:** Summary of pairwise comparisons between the audiovisual Angle offsets (i.e., between the auditory source and the visual representation of the ECA) and the display Device combinations with *Accuracy* as the dependent variable.

Comparison		Median	95% CI	PD	% in ROPE
Angle	Device				
$0^{\circ }-15^{\circ }$	Monitor	-0.01	[-0.04, 0.01]	83.88%	72.15%
$0^{\circ }-30^{\circ }$	Monitor	-0.02	[-0.04, 0.01]	91.64%	56.24%
$0^{\circ }-60^{\circ }$	Monitor	-0.03	[-0.06, -0.01]	99.69%	11.01%
$15^{\circ }-30^{\circ }$	Monitor	-5.13*e*^−3^	[-0.03, 0.02]	65.78%	89.24%
$15^{\circ }-60^{\circ }$	Monitor	-0.02	[-0.05, 0.00]	95.71%	45.07%
$30^{\circ }-60^{\circ }$	Monitor	-0.02	[-0.04, 0.01]	90.73%	61.86%
$0^{\circ }-15^{\circ }$	HMD	9.44*e*^−3^	[-0.02, 0.03]	76.80%	81.20%
$0^{\circ }-30^{\circ }$	HMD	2.77*e*^−3^	[-0.02, 0.03]	50.84%	93.01%
$0^{\circ }-60^{\circ }$	HMD	-7.33*e*^−3^	[-0.03, 0.02]	72.30%	86.04%
$15^{\circ }-30^{\circ }$	HMD	-9.14*e*^−3^	[-0.03, 0.02]	77.12%	82.05%
$15^{\circ }-60^{\circ }$	HMD	-0.02	[-0.04, 0.01]	91.25%	61.02%
$30^{\circ }-60^{\circ }$	HMD	-7.67*e*^−3^	[-0.03, 0.02]	72.99%	86.01%

CI = Bayesian credible interval, PD = probability of direction, ROPE = region of practical equivalence

For *RT_mean* as the dependent variable, similar to *Accuracy*, the final model included all the independent variables and an interaction term between the audiovisual offset angle and display device. However, none of the pairwise comparisons were statistically meaningful with PPDs with low PD values and high % in ROPE (see Table 1 in S3 File), and are not considered further.

#### Questionnaire.

**Fig 5:**
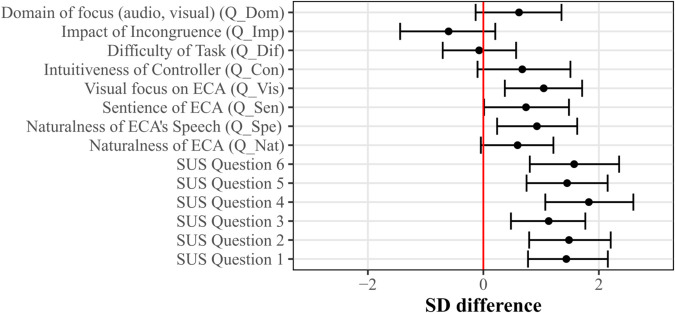
Questionnaire results of Experiment 1. The standard deviation (SD) difference between the HMD and the Monitor display conditions is shown for each question on the y-axis. Error bars indicate the 95% credible intervals. All questions were rated on a scale of 1 to 7, except for Q_Inc, which was asked on a yes/no basis and is thus not displayed here.

The full questionnaire data is given in S5 File. As seen in Fig 5, for all the questions from the SUS questionnaire, the differences in SD between the HMD and computer monitor display conditions were statistically robust, indicating that the participants reported a higher perceived presence in the HMD condition. This is depicted by the 95% CI of the SD difference between display conditions not crossing the zero line, suggesting a meaningful effect.

For the other questions, the 95% CI crosses the zero line, indicating that the SD differences of the ratings between display devices were not statistically robust. This holds true for the naturalness of the ECA (Q_Nat), the extent to which the ECA was perceived as a sentient being (Q_Sen), the intuitiveness of the controller handling (Q_Con), the perceived difficulty of the task (Q_Dif), and the perceived impact of the incongruence on the participants’ performance (Q_Imp).

Four of the 23 participants reported not noticing the audiovisual angle incongruence (Q_Inc). The change in the log odds of the perceived audiovisual incongruence (Q_Inc) between the monitor and HMD condition (-1.14) was not statistically robust with the 95% CI ([-4.63, 1.74]) crossing zero. This indicates that the perceived noticeability of the incongruence did not differ between the display devices.

Statistically robust SD differences between ratings of the display devices could be detected for the naturalness of speech (Q_Spe), with a higher rating of naturalness in the HMD condition, and in the visual focus (Q_Vis), where participants reported that they focused significantly more on the ECA in the HMD condition.

### Discussion

The participants’ avVSR performance was evaluated in terms of *Accuracy* and *RT_mean* for both HMD and computer monitor presentations. No statistically robust influence of the display device on either metric could be found, which is in accordance with the participants’ self-assessment that the task difficulty was similar for both display devices. This suggests that the avVSR task can be successfully conducted in VR, at least in conditions close to the given setup. However, while the SUS questionnaire data indicated a significantly higher perceived presence in the HMD condition, this increased presence did not translate into changes in cognitive task performance. This finding highlights that higher perceived presence in VR does not necessarily correspond to enhanced performance in cognitive tasks.

Regarding the impact of audiovisual angle incongruence, a marginal increase in recall performance was observed for an offset angle of $60^{\circ }$ in the computer monitor presentation, albeit with a negligible effect size. This is quite interesting as a contrary effect, i.e., a decrease in performance, could have been expected. However, it has been shown before that more demanding tasks can evoke increased performance [57], presumably because participants put in more effort. A similar effect may have occured here, i.e., audiovisual angle incongruence might have had an alerting effect, leading to higher concentration and, thus, improved performance. However, we can only speculate as to why this effect occurred in the computer monitor, but not in the HMD condition. As indicated in the questionnaire (Q_Vis), participants focussed more on the visual signal, the ECA, in the HMD than in the computer monitor condition. It is possible that the visual stimulus dominated over the auditory signal and thus the audiovisual angle incongruence could be ignored better. Furthermore, HMDs influence sound source localization [58] and depth perception [59]. This could have impacted the perception of the audiovisual angle incongruence as well. Since the effect size was rather small, further investigations are necessary to validate it.

For all other combinations of display device and audiovisual angle incongruence, no effect of incongruence on recall performance and response time was detected. This is consistent with participants’ ratings, which indicated that they felt only marginally influenced by the audiovisual angle incongruence. One possible explanation for the lack of interference is that participants could largely ignore the incongruence, perhaps because the task-relevant information was primarily auditory, with visual information being redundant.

It is worth noting that the audiovisual incongruence could also be interpreted as a visual distractor. There are only very few studies on the impact of visual distractors on verbal serial recall and visual signals have not been shown to impact recall performance. Liebl *et al*. [60] investigated relatively rapid changes in workspace lighting, namely the effects of a traveling beam on a projector screen in the participants’ field of vision, and found no effect on the visual-verbal serial recall performance. In an experiment with flashing color stripes during stimulus presentation, Lange [61] found no reduction in performance for visual-verbal serial recall. To the best of the authors’ knowledge, auditory-verbal serial recall has not been investigated with visual distractors yet. However, since the ineffectiveness of visual distractors on verbal serial recall is argued to be due to the separation of auditory-verbal and visual-spatial short-term memory according to the model of Baddeley and Hitch [62], it is reasonable to assume that the audiovisual incongruence did not act as a performance-relevant distractor in the present study with auditory reproduction either. In summary, our results suggest that participants are able to ignore audiovisual angle incongruence in avVSR tasks, at least in experimental settings resembling the current.

The ratings regarding the general appearance of the ECA did not differ between display devices in a statistically robust way. Similar effects regarding ECAs can be found in literature. McKeown *et al*. [63] conducted a study where participants rated social interactions presented either on an HMD or a computer monitor. These ratings were not influenced by the display device. In a study on human-ECA interactions, Zojaji *et al*. [64] found no influence on the perceived persuasiveness and offensiveness between display devices. This suggests that the perception of an ECA may not generally be influenced by the display device. Interestingly, in the present study, the ECA’s speech was perceived as more natural in the HMD condition compared to the computer monitor condition, although the exact same material (and in the case of the speech even audio reproduction) was used. This may be linked to the higher presence overall and higher visual focus reported in the HMD condition.

The experiment had several limitations for both display devices. Firstly, with the employed animation technique (computer-generated lip movement, no blinking, and only idle movement), the subjective evaluation of the naturalness of the ECA was generally low. Based on the general comments, the participants found the ECA’s eye movement, or rather the lack thereof, particularly unnatural, which provided an opportunity for improvement. Secondly, regarding the choice of incongruence, the behavioral results indicate that participants were able to ignore it. According to the questionnaire, a total of 13% of participants did not notice the audiovisual angle incongruence at all. It is possible that the selected angle differences were insufficiently large, and that only the $60^{\circ }$ condition entered the range of disruptive incongruences. We derived the selection of angles from ventriloquism experiments, in which participants focus on the spatial characteristics of the stimuli since their task is to localize them. In contrast, the perception of spatial direction was incidental in our study, as subjects were instructed to focus on the content, specifically the to-be-remembered digits. This could have shifted the threshold for detection, and/or the need to attend to incongruences. Also, participants were likely experienced in integrating audiovisual angle incongruences. For example, when watching a movie, no directional discrepancy between audio and video is perceived actively, even though loudspeakers are usually placed beside the screen. It remains unclear if the participants were able to ignore the incongruence due to the small angle range and the familiarity of the situation - or if incongruent audiovisual stimuli can be ignored in general if they are not task-relevant.

## Experiment 2: Audiovisual semantic incongruence

A limitation of Experiment 1 was that the spatial incongruence went unnoticed by many participants, potentially confounding the findings on audiovisual incongruences in VR. Moreover, the chosen angle incongruence made it challenging to distinguish between the transition from congruent to incongruent cases, as the angle is a continuous variable with no clear threshold for noticeability. To address this issue, a clearly noticeable audiovisual incongruence was considered in Experiment 2. One potential approach to ensure perceptibility is a pre-test with each participant to determine individual angular thresholds of detectibility, similar to the study by Kim and Lee [20]. However, even with this refined approach, we could not be sure that the participants would interpret the spatial incongruence as such. As discussed previously, most people naturally integrate spatial incongruent audiovisual stimuli, especially in entertainment applications. Therefore, we wanted to use a binary and more easily distinguishable incongruence which allows for clearly comparing congruent with incongruent cases.

**Fig 6:**
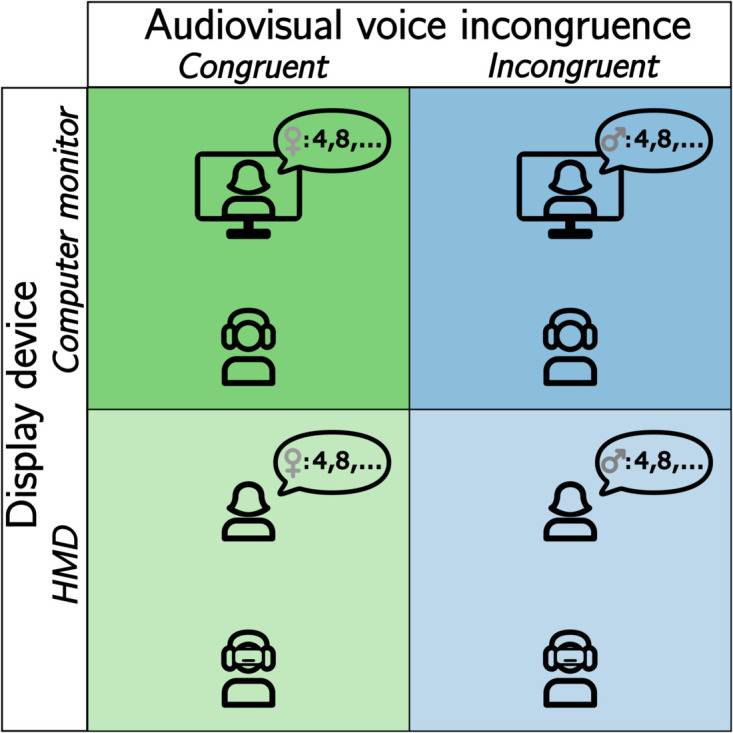
Factors in Experiment 2. The between-subject experimental design of the avVSR task with three factors: display device (computer monitor vs. HMD), the audiovisual voice incongruence (congruent vs. incongruent), and the serial position (1 – 8) of the target digit sequence. In the congruent case, the ECA speaks with their own voice; in the incongruent case the female ECA speaks with the male ECAs’ voice and vice versa (here shown for the female ECA).

For this purpose, in Experiment 2, a voice swap, i.e., two ECAs speaking with each others’ voices, was introduced. To allow a clear distinction between the voices, a swap between a male and female ECA was chosen. A voice swap can be considered as a special case of semantic incongruence because it disrupts the expected auditory-visual correspondence, leading to a mismatch between the perceived voice and the visual identity or context. This *audiovisual voice incongruence* was investigated in both HMD and computer monitor presentations. An overview over the experimental design can be found in Fig 6. Compared to Experiment 1, the position of the ECA was not changed in this experiment, and hence no spatial incongruence was included.

### Method

#### Participants.

The recruitment procedure, screening, ethics approval, consent form, and compensation were the same as in Experiment 1. Participants were recruited between 30th of November and 23rd of December 2022. *N* = 26 adults (16 male, 10 female) aged between 21 and 39 years (M=28.08,SD=4.39) participated in Experiment 2. It was not an exclusion criterion if participants had already participated in the first study.

#### Cognitive task.

The paradigm for the cognitive task was the same as in Experiment 1: an avVSR task in which digit sequences were played back auditorily and an ECA was animated to speak the sentences (see Fig 2).

#### Audiovisual voice incongruence.

**Fig 7:**
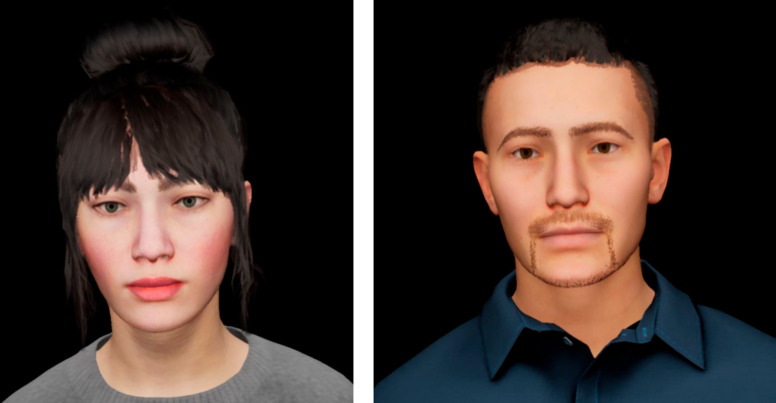
ECAs used in Experiment 2. *Left:* The female ECA was the same as in Experiment 1. *Right:* A male ECA was created for Experiment 2.

A voice swap was implemented as the audiovisual incongruence. At the beginning of the experiment, two ECAs one female and one male (see Fig 7), were introduced with their respective voices. In the experiment, they unexpectedly spoke with the voice of the other ECA, thus, swapping voices for an audiovisual incongruence (male-female, female-male). Voice swaps have been used in cognitive research before. Walker-Andres *et al*. [65] found that infants as young as six months prefer to look at audiovisual stimuli with congruent gender compared to incongruent gender. In an experiment of just noticeable onset asynchronies of audiovisual stimuli, Vataksi and Spence [66] found a higher sensitivity for gender-incongruent stimuli. They suggested that a incongruence between the auditory and visual stimuli in terms of gender leads to them not being perceived as a unit. A study by Szerszen [67] indicated that a gender incongruence in the voice can lead to an uncanny valley effect. In this experiment, the position of both the auditory and visual sources was kept constant at an azimuth angle of $\varphi =0^{\circ }$ on the horizontal plane to avoid the additional effect of spatial incongruence.

#### Apparatus and materials.

The same equipment and software as in Experiment 1 were used. A second, male, ECA was created. Since participants reported that the gaze of the ECA seemed unnatural in Experiment 1, an idle eye movement was added for both ECAs using the VHGazeComponent of the Character Plugin in Speech Mode (for more information see [68]). The male voice stimuli were taken from [40] (voice *male a*).

#### Questionnaires.

The questionnaires remained unchanged from Experiment 1.

#### Procedure.

The procedure was mainly the same as in Experiment 1. The training block consisted of six trials (three with the female ECA, three with the male ECA) with matching voices. This way, participants could familiarize themselves with the task and the voice matches. In the main experiment, twelve trials were presented for all four possible combinations of the ECAs and their voices, resulting in 12×4=48 trials per display device, divided into four blocks of twelve trials each. The order of the visual ECA and voice combinations was counterbalanced across participants in each block.

#### Statistical analysis.

The procedure for the statistical analysis was identical to that in Experiment 1 except for two changes. First, audiovisual voice incongruence was introduced as a binary independent variable. The other two independent variables were the same as in Experiment 1 (display device and serial position), with the dependent variables also being the same (*Accuracy* and *RT_mean*). Secondly, the priors for display device and serial position were set based on the respective PPD from models in Experiment 1.

### Results

#### Audiovisual verbal serial recall.

The full result data of Experiment 2 is given in S2 File, plots of the data can be found in S3 File. Similar to Experiment 1, the leave-one-out criteria was used to determine the final models. For *Accuracy* as the dependent variable, the model with all the IVs and an interaction term between the audiovisual voice incongruence and display device was selected as the final model since it encompasses the experimental design most comprehensively. The pairwise comparisons for this model are presented in Table 2. While the PD for the Monitor condition was greater than 97.5% (equivalent to a *p*-value of less than .05), suggesting better performance for incongruent compared to congruent trials, neither of the comparisons here was statistically meaningful with the % of the PPD within ROPE being very high.

**Table 2:** Summary of pairwise comparisons between the voice incongruence (Match) and display Device combinations with *Accuracy* as the dependent variable.

Comparison		Median	95% CI	PD	% in ROPE
Match	Device				
Incong.–Cong.	Monitor	0.02	[ 0.00, 0.04]	97.71%	58.72%
Incong.–Cong.	HMD	0.01	[ 0.00, 0.03]	94.80%	72.24%

CI = Bayesian credible interval, PD = probability of direction, ROPE = Region of practical equivalence, Incong. = Incongruent, Cong. = Congruent

For *RT_mean* as the DV, the final model also included all the independent variables and an interaction term between audiovisual voice incongruence and display device. However, as in Experiment 1 and for *Accuracy* as the dependent variable in this experiment, none of the pairwise comparisons were statistically meaningful (see Table 2 in S3 File), and are not considered further.

#### Questionnaire.

**Fig 8:**
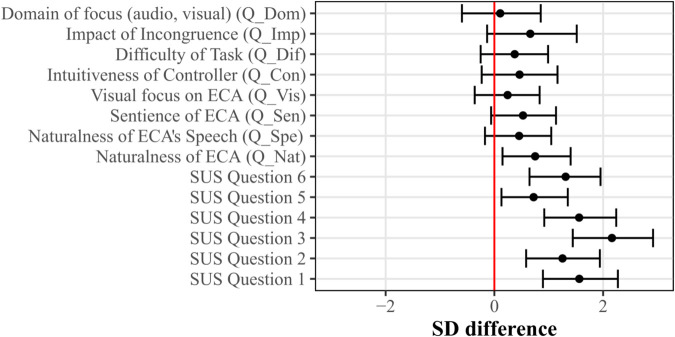
Questionnaire results of Experiment 2. The standard deviation (SD) difference between the HMD and the Monitor display conditions is shown for each question on the y-axis. Error bars indicate the 95% credible intervals. All questions were rated on a scale of 1 to 7, excluding Q_Inc, which was asked on a yes/no basis and is thus not displayed here.

All questionnaire data is given in S5 File. The results of the questionnaire analysis are displayed in Fig 8. As in Experiment 1, the SD differences between the ratings on the SUS questionnaire for the two display conditions were statistically robust (95% CI does not include zero), with the participants reporting higher presence in the HMD vs. the computer monitor condition. The ECAs’ naturalness (Q_Nat) was rated higher in the HMD condition.

For the remaining questions, the SD differences between ratings for the conditions were not significantly robust. Only one participant reported not noticing the voice incongruence (Q_Inc). However, the change in the log odds of the perceivability of the voice incongruence (Q_Inc) between the monitor and HMD condition (–0.01) was not statistically robust with the 95% CI ([–4.01, 3.99]) crossing zero. This suggests that the perception of incongruence did not vary across the devices.

**Fig 9:**
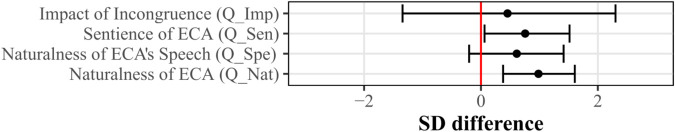
Standard deviation (SD) difference of questionnaire data between Experiment 1 and 2. Results are shown for the questions Q_Nat, Q_Sen, Q_Spe, and Q_Imp (on the y-axis). Error bars indicate the 95% credible intervals. All questions (see section *Questionnaire*) were rated on a scale of 1 to 7.

Finally, the questionnaire results from Experiment 1 were compared with the results from Experiment 2 to see if the changes in the experimental design (changing the type of audiovisual incongruence, adding eye movements to the ECA animation) had any effect. To that end, the subjective impact of the two types of audiovisual incongruences (Q_Imp) and the three questions related to the ECA (Q_Nat, Q_Spe, Q_Sen) were compared between Experiment 1 and Experiment 2 using the same analysis procedure as for the individual questionnaires with the experiment as the dependent variable. The results are displayed in Fig 9. Indeed, statistically relevant differences in terms of perceived naturalness (Q_Nat) and sentience (Q_Sen) were found between Experiments 1 and 2, with higher ratings in Experiment 2. Differences in the naturalness of speech (Q_Spe) and the perceived impact of the incongruence (Q_Imp) were not statistically robust.

### Discussion

In Experiment 2, task performance in terms of *Accuracy* and *RT_mean* in the serial recall were examined both for HMD and computer monitor presentations. As in Experiment 1, no meaningful influence of the display device on either performance metrics could be found. This again suggests that the avVSR task can be successfully conducted in VR, at least in conditions close to the given setup.

In this experiment, we used a more obvious and binary audiovisual distractor by introducing an audiovisual voice incongruence as a type of semantic incongruence. This reduced the number of participants who failed to notice the incongruence from 13 In Experiment 1, where audiovisual angle incongruence was investigated, a small increase in recall performance could be found for the audiovisual offset angle of $60^{\circ }$ in the computer monitor presentation. We hypothesized this to be due to the alerting effect of the incongruence. In the current experiment employing the audiovisual voice incongruence, again, the *Accuracy* was slightly increased in trials with incongruent voice and the computer monitor as display device. However, this finding was not statistically robust. Even though the type of incongruence was fundamentally different between the experiments, it is reasonable to argue that *Accuracy* is not affected by these incongruences in a statistically relevant way. In other words, the cognitive performance in the avVSR task seems robust against the two incongruences that were tested. While our findings contribute to the understanding of the complex mechanisms underlying the processing of audiovisual stimuli, further research is needed to comprehensively explore the intricate interactions and neural pathways involved.

As in Experiment 1, increased presence could be detected in Experiment 2 for the HMD presentation in the questionnaires. In Experiment 1, the naturalness of speech of the ECA was rated higher in the HMD condition and the ratings of the general naturalness of the ECA was not affected by the display device. This was the other way around in Experiment 2, where the ratings of the naturalness of speech were not affected by the display device, but the general naturalness of the ECA was rated higher in the HMD condition, even though the same lip animation algorithm and speech material were used. This indicates that while the display device does influence the perception of an ECA, the specific nature and nuances of this influence remain unclear. The improved animation of the ECA led to better ratings with regard to naturalness and the degree to which the ECA is perceived as a sentient being compared to Experiment 1. Adding even more animations to the ECA, like gestures and plausible mimics, could further increase perceived naturalness. It should be noted that participants’ ratings were based on their perception of the scene while performing the memory task, which may not be generalizable to other contexts. Still, since differences in the display device became apparent only in the questionnaire and not in the performance data, our study highlights the need for a multidimensional evaluation framework when assessing VR environments.

## Limitations

It is important to note that both the experiments were conducted under relatively simple conditions - specifically, a minimalistic and static laboratory scene featuring only the conversing ECAs as an additional element. Coherent audiovisual cues are likely to play a more critical role when focusing on a target source becomes more difficult in more complex and busier scenes. To gain further insights into how incongruences affect participants, future research should consider increasing the complexity of the scenes by introducing acoustic distractors (such as background noise) and visual distractors (like additional embodied conversational agents or moving scene objects) that bear increasing resemblance to real-life situations. In a busier scene with multiple distractions, participants may find it increasingly challenging to extract and attend to the voice of an ECA if it does not match its visual representation (cp. Experiment 2) or if it is perceived from a different direction (cp. Experiment 1). Another limitation was that no a-priori power calculations were conducted, and the sample size may be considered relatively small. The Bayesian analysis used here can be robust against smaller sample sizes when the priors are informative and the model is specified correctly [69]. The priors used here were, however, weakly/mildly informative since effect sizes from previous studies (not compatible enough with the current experimental design) could not be incorporated within informative priors. Hence, arguably larger sample sizes may be necessary in future studies that can at least benefit from the findings here.

## Conclusion

Two experiments were conducted to investigate the influence of audiovisual spatial (angle) and semantical (voice) incongruence, simulated over two display devices (HMD and computer monitor), on avVSR performance and subjective ratings. With regard to audiovisual incongruences, no clear effect on the porportion of correctly recalled items (*Accuracy*) or response times (*RT_mean*) could be detected. Some tendencies of increased performance were observed within the computer monitor presentation for the more extreme angle incongruences. The only difference between display devices could be found in the SUS questionnaire, which consistently revealed higher presence when using an HMD compared to a computer monitor in both experiments. Ratings of the naturalness and sentience of the ECAs did not change with the display device.

So far, research on audiovisual incongruence has mostly focused on the perception level, whereas this paper investigated its relevance for verbal short-term memory, which has not been studied previously. Generally speaking, participants were able to ignore the types of audiovisual incongruences presented in the avVSR task. Further research is needed to explore the relation between perceptibility and task impact in more complex scenes, which could provide further insights into the role of audiovisual congruence in cognitive processing and VR design.

## Supporting information

S1 File Data of Experiment 1. *Accuracy* and *RT_mean* for each condition and serial position in Experiment 1 (audiovisual angle incongruence).
(CSV)

S2 File Data of Experiment 2. *Accuracy* and *RT_mean* for each condition and serial position in Experiment 2 (audiovisual voice incongruence).
(CSV)

S3 File Data plots and analysis of RT_mean Experiment 1 and 2. Plots of *Accuracy* and *RT_mean* as well as results of the statistical analysis of *RT_mean* for Experiment 1 and 2.
(PDF)

S4 File Questionnaire. Questions asked in the questionnaires (except for SUS questions) with corresponding anchors.
(TXT)

S5 File Questionnaire Data Experiment 1 and 2. Ratings for all questions of the questionnaire for Experiments 1 and 2.
(CSV)
