# Supplementary material for: Audiovisual angle and voice incongruence do not affect audiovisual verbal short-term memory in virtual reality
Source: PLoS One. 2025 Aug 22;20(8):e0330693. doi: 10.1371/journal.pone.0330693 (PMC12373245; doi:10.1371/journal.pone.0330693)
Supplement: S3 File — Plots of Accuracy and RT_mean as well as results of the statistical analysis of RT_mean for Experiment 1 and 2. (PDF) [file pone.0330693.s003.pdf]

Supplementary File S3 for the paper:

Audiovisual angle and voice incongruence do not affect audiovisual verbal short-term memory in virtual reality

Cosima A. Ermert[1], Manuj Yadav[1], Jonathan Ehret[2], Chinthusa Mohanathasan[3], Andrea Bönsch[2], Torsten W. Kuhlen[2], Sabine J. Schlittmeier[3], Janina Fels[1]

1 Institute for Hearing Technology and Acoustics, RWTH Aachen University, Aachen, Germany

2 Visual Computing Institute, RWTH Aachen University, Aachen, Germany

3 Work and Engineering Psychology, RWTH Aachen University, Aachen, Germany

* cosima.ermert@akustik.rwth-aachen.de (CE)

Plots of mean results of Experiment 1 and 2

In the following, the data of Experiment 1 (angle incongruenc) and Experiment 2 (voice incongruence) are displayed.

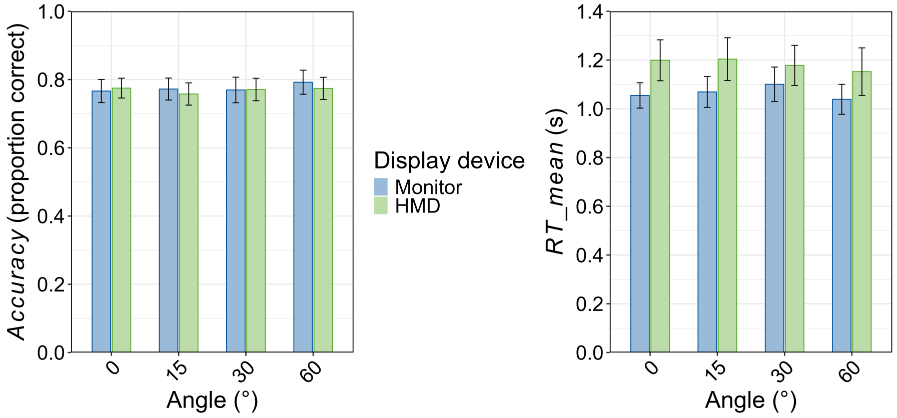

Fig 1. ***Accuracy* in terms of proportion correct and mean response time *RT_mean* in Experiment 1 (angle incongruence). The error bars represent the standard error of the mean.**

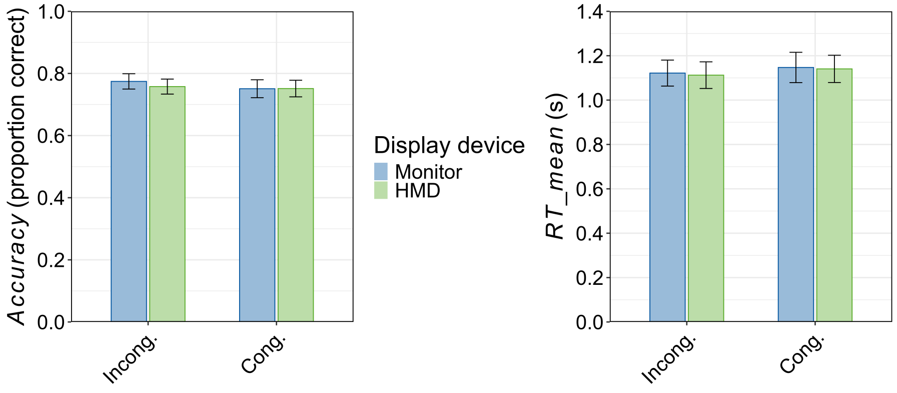

Fig 2. ***Accuracy* in terms of proportion correct and mean response time *RT_mean* in Experiment 2 (voice incongruence). The error bars represent the standard error of the mean.**

Results of statistical analysis of the average response time *RT_mean*

For details regarding the statistical analysis, please refer to the main paper. The final statistical model of the dependent variable (DV) *RT_mean* included the independent variables serial position, audiovisual angle or voice incongruence, and display device, as well as an interaction term between the audiovisual angle incongruence and the display device. The interaction term with serial position was not considered useful for the research intend and hence not included. None of the pairwise comparisons were statistically meaningful with PPDs with low probability of direction (PD) values and high % in region of practical equivalence (ROPE).

Table 1. Summary of pairwise comparisons between the audiovisual angle incongruences and display device combinations with *RT_mean* as the DV (Experiment 1).

Comparison		Median	95% CI	PD	% in ROPE
Angle	Device				
0° − 15°	Monitor	$7.51e^{-3}$	[-0.05, 0.07]	59.40%	100%
0° − 30°	Monitor	-0.01	[-0.07, 0.05]	68.01%	98.61%
0° − 60°	Monitor	0.03	[-0.03, 0.09]	83.40%	91.74%
15° − 30°	Monitor	-0.02	[-0.08, 0.04]	75.78%	95.94%
15° − 60°	Monitor	0.02	[-0.04, 0.08]	77.38%	95.53%
30° − 60°	Monitor	0.04	[-0.02, 0.11]	92.64%	80.04%
0° − 15°	HMD	-0.02	[-0.08, 0.05]	67.97%	97.30%
0° − 30°	HMD	0.03	[-0.04,0.09]	81.16%	91.15%
0° − 60°	HMD	0.05	[-0.01, 0.11]	93.69%	76.09%
15° − 30°	HMD	0.04	[-0.02, 0.11]	91.10%	79.43%
15° − 60°	HMD	0.06	[0.00, 0.13]	97.32%	57.92%
30° − 60°	HMD	0.02	[-0.04,0.08]	72.97%	96.76%

CI = Bayesian credible interval, PD = probability of direction, ROPE = region of practical equivalence

Table 2. Summary of pairwise comparisons between the voice incongruence and display device combinations with *RT_mean* as the DV (Experiment 2).

Comparison		Median	95% CI	PD	% in ROPE
Match	Device				
Incong.−Cong.	Monitor	-0.03	[-0.08, 0.02]	87.09%	96.61%
Incong.−Cong.	HMD	$-9.60e^{-3}$	[-0.06, 0.04]	64.46%	100%

CI = Bayesian credible interval, PD = probability of direction, ROPE = Region of practical equivalence, Incong. = Incongruent, Cong. = Congruent
